# Cerebral Venous Thrombosis:Current Status and Challenges

**DOI:** 10.1002/brb3.70844

**Published:** 2025-09-09

**Authors:** Wenbo Zuo, Shengcai Chen, Jiashuo Lin, Yan Wan, Fei Cao, Bo Hu

**Affiliations:** ^1^ Department of neurology, Union Hospital, Tongji Medical College Huazhong University of Science and Technology Wuhan China

**Keywords:** cerebral venous thrombosis, sinus thrombosis, prognosis, risk factor, treatment

## Abstract

**Background:**

Cerebral venous thrombosis (CVT) is a stroke type that primarily affects young individuals, with various risk factors and complex mechanisms. It accounts for 0.5% to 3% of all stroke cases and can significantly impact daily activities and quality of life.

**Objective:**

This review aims to classify the etiology of CVT, assess its impact on prognosis, and evaluate the best treatment approaches. It also analyzes current issues in CVT management and proposes future directions for treatment.

**Methods:**

A comprehensive review of the literature on CVT, focusing on risk factors, treatment strategies, and outcomes. The impact of anticoagulation therapy and endovascular interventions is examined.

**Results:**

This review examines the various risk factors associated with CVT, including female‐specific conditions, infections, autoimmune diseases, cancer, and mechanical triggers. It highlights the impact of these factors on the prognosis and outcomes of CVT. The effectiveness of anticoagulation therapy, both traditional and with direct oral anticoagulants, is discussed, along with the role of endovascular therapy in severe cases. The review also identifies current challenges in CVT management, such as incomplete thrombus clearance and the lack of standardized evaluation criteria for recanalization.

**Conclusion:**

Further research is needed to optimize CVT management, improve patient outcomes, and enhance the quality of life for affected individuals.

## Graphical abstract

Our review introduces treatment strategies for short‐term, long‐term, and permanent CVT risk factors, focusing on anticoagulant selection and treatment duration. We also discuss the current status of endovascular therapy for CVT, noting that it remains a challenge due to limited evidence, and more research is needed to optimize this approach.

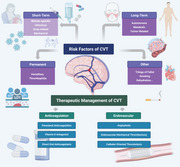

## 1 Introduction

Cerebral venous thrombosis (CVT) is a relatively rare but potentially serious disease characterized by the presence of blood clots within the dural venous sinuses or cerebral veins. CVT represents a notable subset of stroke cases, accounting for 0.5% to 3% of all strokes (Alet et al. 2020). In recent population‐based studies, the incidence of CVT has been identified as 1.32 to 1.57 per 100,000 person‐years. Despite its lower prevalence compared with stroke, CVT can affect individuals of any age, predominantly younger adults, with a significant proportion occurring in individuals under the age of 55 (Saposnik et al. 2024). Women are more prone to CVT, and the incidence rate is two or three times that of men, which is mainly due to the influence of female hormones (Aamodt and Skattør 2022).

Risk factors for CVT are diverse, including women on long‐term oral contraceptives, those who are pregnant or in the postpartum period, individuals with autoimmune diseases, thrombophilia, cancer, concurrent infections, as well as those who have undergone surgery, lumbar puncture, or experienced trauma (Duman et al. 2017; Ferro et al. 2004). Whether the etiology is controllable is the key factor to determine the prognosis. Also, thrombus burden, without standardized method for assessment, is closely related to the severity of the disease, and its residue has been linked to persistent neurological sequelae, including headaches, neuropsychological issues, and visual impairments (Ferro and Aguiar De Sousa 2019; Putaala, Hiltunen, Salonen, Kaste, and Tatlisumak 2010; Wang et al. 2023).

Anticoagulation therapy is the cornerstone of CVT treatment, typically lasting from 3–12 months to lifelong, irrespective of the underlying cause (Saposnik et al. 2024). This therapy, however, increases the risk of bleeding complications, with major bleeding rates of 2.44–4.70 per 100 patient‐years and intracranial hemorrhage rates of 1.52–3.51 per 100 patient‐years (Yaghi et al. 2022). Despite anticoagulation, recurrent venous thromboembolism occurs in approximately 5.26–5.87 per 100 patient‐years, and about 15% of patients exhibit persistent vessel occlusion with residual thrombi (Yaghi et al. 2022). Endovascular therapy is considered a second‐line option for CVT patients whose symptoms worsen despite anticoagulation therapy, particularly for those with severe conditions such as neurologic deterioration, altered mental status, coma, intracranial hemorrhage, or deep venous thrombosis (Coutinho et al. 2020). The effectiveness of this approach in improving the long‐term outcomes for these patients is still a matter of debate.

While 68–80% of patients under anticoagulation therapy achieve a modified Rankin Scale (mRS) score of 0–1 at 12 months, indicating favorable outcomes, the mRS score could not evaluate the residual symptoms such as headaches, fatigue, neurocognitive deficits, or seizures, (Coutinho et al. 2020; Dentali et al. 2012; Jedi et al. 2022; Le et al. 2024; Siddiqui et al. 2019; Zhou et al. 2024) which can significantly impair daily activities and quality of life, even in the absence of severe physical disability (de Bruijn, Budde, Teunisse, de Haan, and Stam 2000; Hiltunen et al. 2016; Koopman et al. 2009).

This review summarizes the latest evidence of risk factors for CVT, and clearly distinguishes between clinically controllable factors such as oral contraceptives, infection control or perioperative anticoagulation and basically unalterable factors, including hereditary thrombosis or chronic autoimmune diseases. It provides a critical appraisal of conventional anticoagulation strategies versus emerging direct oral anticoagulants (DOACs) for CVT, alongside a systematic analysis of contentious endovascular interventions—mechanical thrombectomy, catheter‐directed thrombolysis, and angioplasty, trying to address unresolved questions of patient selection, timing, and comparative efficacy. By proposing novel insights into thrombus‐burden quantification and long‐term, patient‐centered outcomes beyond mRS, the article offers clinicians and researchers an updated roadmap for optimizing individualized CVT management and future trial design.

## 2 Method

The PubMed database was searched using keywords “cerebral venous thrombosis,” “cerebral vein thrombosis,” “dural sinus thrombosis,” “intracranial sinus thrombosis,” and “cavernous sinus thrombosis.” Supplementary manual searches of reference lists of retrieved articles were conducted to avoid missing eligible studies. Included were studies on CVT epidemiology, risk factors, pathophysiology, clinical features, diagnosis, and treatment—covering randomized controlled trials, cohort studies, case‐control studies, systematic reviews, meta‐analyses, and clinically significant case reports, with participants having confirmed CVT (including adults, children, pregnant women, and other populations).

## 3 Risk Factors of CVT

**FIGURE 1:**
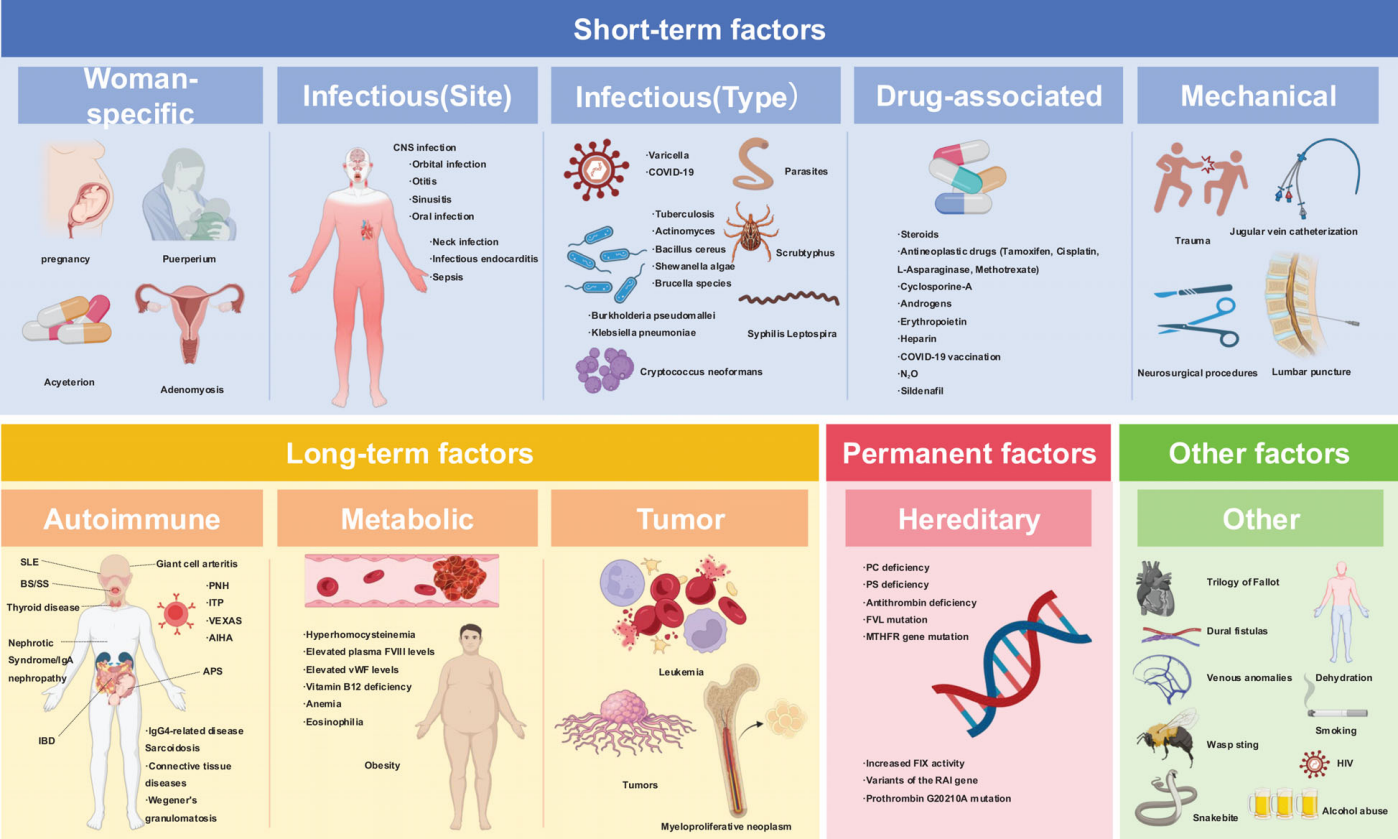
Classification of risk factors for cerebral venous thrombosis (CVT). CVT risk factors are categorized into four groups: short‐term factors, including woman‐specific factors, infections (by site and type), drug‐associated factors, and mechanical factors; long‐term factors, mainly autoimmune diseases, metabolic abnormalities, and tumors; permanent factors, primarily hereditary thrombophilia; and other unclassified influencing factors. Abbreviations; vWF, von Willebrand factor; FVIII, factor VIII; N2O, nitrious oxide; PC, protein C; PS, protein S; FVL, factor V Leiden; MTHFR, methylenetetrahydrofolate reductase; FIX, factor IX; RAI, retinoic acid‐induced; SLE, systemic lupus erythematosus; BS, Behçet's syndrome; SS, Sjögren's syndrome; IBD, inflammatory bowel disease; PNH, paroxysmal nocturnal hemoglobinuria; ITP, immune thrombocytopenia; VEXAS, (vacuoles, E1 enzyme, X‐linked, autoinflammatory, and somatic syndrome); AIHA, autoimmune hemolytic anemia; COVID‐19, Corona Virus Disease 2019; HIV, human immunodeficiency virus; CNS, central nervous system.

We summarize CVT risk factors into four tiers (Figure 1) to provide clinicians with an immediate roadmap for identifying actionable targets. Short‐term factors—woman‐specific circumstances, infections (site‐ and pathogen‐defined), drug exposures and mechanical triggers—are largely modifiable and therefore primary candidates for prompt intervention. Long‐term contributors (autoimmune disorders, metabolic derangements and malignancy) are partially modifiable, guiding sustained preventive strategies. Permanent determinants (hereditary thrombophilias) are non‐modifiable and inform prophylactic intensity rather than reversal. A fourth “other” category flags currently unclassified influences awaiting further definition. Each subgroup is discussed below to clarify its mechanistic link to CVT and, more importantly, to highlight where clinical action can improve patient prognosis.

In addition, we have consolidated all risk factors and their evidence‐based countermeasures into a single comparative table (Supplementary Table 1).

### 3.1 Temporary Risk Factors

#### 3.1.1 Woman‐Specific Factors

Marked sex‐based disparities in CVT incidence are evident, with females demonstrating a twofold higher incidence than males (15.1 vs. 7.8 per million population) and a rising trend (2002: 13.4 vs. 2012: 20.5 per million) (Rezoagli et al. 2021). Risks are particularly elevated during the perinatal period, showing a 3.8‐fold increase in pregnancy, escalating to 10.6‐fold post‐delivery and peaking at 18.7‐fold within 6 weeks postpartum (Silvis et al. 2019). A systematic review of 217 pregnant patients with CVT history across 13 studies demonstrated 16–80‐fold higher recurrence rates than the general population,(Aguiar de Sousa et al. 2016), highlighting the necessity for targeted preventive strategies in high‐risk female cohorts.

CVT diagnosis presents challenges due to nonspecific manifestations such as positional headaches, visual disturbances, seizures, and focal deficits (Wasay, Kojan, Dai, Bobustuc, and Sheikh 2010), with physical examination potentially revealing papilledema or meningeal irritation (Marwah et al. 2016). In perinatal populations, thorough evaluation of medical history and neurological status is required, supplemented by hematological indicators like elevated D‐dimer. Neuroimaging protocols prioritize non‐contrast MRI/MRV techniques (2D time‐of‐flight/3D phase‐contrast venography) to minimize fetal radiation exposure, while contrast‐enhanced MRV remains limited to specific scenarios with cautious gadolinium use (Durmuș et al. 2020; Ferro and Aguiar De Sousa 2019). Susceptibility‐weighted imaging differentiates acute/subacute thrombi, complemented by DWI for late‐subacute phase identification. CT venography serves as a secondary option when MRI is contraindicated, despite reduced sensitivity for cortical vein detection (Durmuș et al. 2020). Early recognition of these modifiable risk factors is critical to prevent neurological complications.

Obesity and oral contraceptive use constitute significant CVT risk factors in women, predominantly mediated by hormonal thrombogenic mechanisms (Zuurbier et al. 2016). Estrogen enhances coagulation via endothelial activation, notably through von Willebrand factor (vWF) upregulation and prolonged half‐life, as evidenced in pregnancy and estrogen replacement therapy (Abou‐Ismail et al. 2020). Combined hormonal contraceptives (CHC) induce a procoagulant state by elevating factors II, VII, VIII, X, and fibrinogen, coupled with reductions in factor V, tissue factor pathway inhibitor, and antithrombin (Abou‐Ismail et al. 2020; Cosman et al. 2005). Although CHC increase protein C activity, concurrent rises in α1‐antitrypsin/α2‐macroglobulin and decreased protein S antigen negate this protective effect (Abou‐Ismail et al. 2020; Skeith and Bates 2024). CHC‐mediated fibrinolytic alterations—characterized by decreased plasminogen activator inhibitor‐1 (PAI‐1) and elevated tissue plasminogen activator (tPA)—show unclear clinical relevance to estrogen‐associated venous thromboembolism (Skeith and Bates 2024).

Gene‐environment interactions further amplify CVT risk in women. Obesity, characterized by elevated prothrombotic factors (PAI‐1, vWF) and activated protein C (APC) resistance, interacts with genetic predispositions such as factor V Leiden mutation to augment clotting tendencies (Christiansen et al. 2012; Zuurbier et al. 2016). Oral contraceptives compound this risk by synergizing with genetic variants to increase APC resistance in a dose‐dependent manner, particularly among individuals with BMI ≥25, where CVT risk rises to 11.87‐fold (BMI > 25) and 29.26‐fold (BMI > 30) (Zuurbier et al. 2016).

Targeted interventions must address these mechanistic pathways. For oral contraceptive users, coagulation monitoring (factor VIII, vWF, APC resistance) may identify high‐risk individuals, while obesity management—focused on reducing inflammatory states and improving coagulation factor profiles—represents a key preventive strategy (Zuurbier et al. 2016). In pregnancy, addressing modifiable risks like immobility, cesarean section‐associated stasis, and preeclampsia‐related hypercoagulability through anticoagulant prophylaxis and close thromboprophylaxis has shown efficacy in reducing recurrence (Algahtani, Bazaid, Shirah, and Bouges 2022). Future research should explore personalized approaches integrating genetic testing with hormonal and metabolic profiling to optimize preventive strategies for high‐risk female populations.

Furthermore, adenomyosis, a relatively common condition among women, has occasionally been reported in cases associated with CVT (Li et al. 2023). The mechanism seems to be related to a hypercoagulable state induced by anemia. Typically, prognosis is favorable with iron supplementation and anticoagulant therapy, and recurrence rates are low. Whether hysterectomy could further reduce recurrence rates remains an area for future research (Li et al. 2023).

In addition, the recurrence rate of women patients with CVT is much less than men, approximately 1.5% ‐3% within 1 year, around 12% within 5 years, and approximately 18% within 10 years (Miranda et al. 2010; Palazzo et al. 2017). Even for pregnant patients with a history of CVT, after receiving heparin treatment, the probability of recurrent venous thrombosis decreases from 4.8% to 1% (Diana Aguiar de Sousa, Canhão, and Ferro 2018), indicating that a history of CVT may not be a contraindication for pregnancy.

#### 3.1.2 Infectious Risk Factors

Apart from female‐specific factors, systemic infections represent another major modifiable risk category, with distinct pathophysiology and management implications. Infection‐related CVT comprises non‐septic thrombophlebitis and septic cerebral venous thrombosis (SCVT), predominantly originating from head/neck infections (otitis media, pharyngitis, sinusitis) (Kojan and Al‐Jumah 2006). While infective endocarditis remains a rare etiology with favorable anticoagulation outcomes despite bleeding concerns (Raharimaminjatovosoa et al. 2022), SCVT incidence and mortality have markedly declined post‐antibiotic era (Kojan and Al‐Jumah 2006). Epidemiological data reveal divergent infection‐related CVT proportions: 47.1% in Northwest Italy (2002–2012) versus 8.1–12.3% in international cohorts (Duman et al. 2017; Ferro et al. 2004), with in‐hospital mortality averaging 3%. Sepsis elevates early mortality risk 7.5‐fold (15.6% vs. non‐septic) (Nasr, Brinjikji, Cloft, Saposnik, and Rabinstein 2013). Pediatric populations exhibit distinct patterns: infection‐related CVT incidence reaches 36.6%, with lower headache prevalence (36.6% vs. adult 90%) and higher mortality (3–12%) (Karakas et al. 2024). Neonates predominantly present with encephalopathy/seizures, whereas children show intracranial hemorrhage, with long‐term sequelae affecting 44.4% and 37.9% respectively (Chtara et al. 2023). Notably, head/neck infections independently predict adverse outcomes in pediatric CVT (Karakas et al. 2024).

While COVID‐19 has heightened awareness of CVT, it does not significantly elevate CVT‐related mortality (Scutelnic et al. 2024). Current evidence supports early anticoagulation (typically 3 months) combined with antiepileptic management for COVID‐19‐associated CVT, though optimal duration requires further validation (Ghosh et al. 2021). Infection‐related CVT management follows pathogen‐specific protocols: 1) Actinomyces infections necessitate surgical debridement with 6–12 months penicillin/amoxicillin and 6‐month anticoagulation to prevent thrombus progression (Martins Sousa et al. 2022); 2) Tuberculosis requires 3–6 months anticoagulation alongside standardized antitubercular therapy (Li et al. 2023); 3) Klebsiella pneumoniae sepsis employs staged antimicrobial regimens (intensive to sequential oral) with rivaroxaban for persistent thrombosis (Zhou et al. 2022); 4) Burkholderia pseudomallei mandates surgical resection followed by 2–6 weeks ceftazidime/meropenem, transitioning to 6‐month cotrimoxazole with concurrent anticoagulation (Bahuleyan et al. 2022). Scrub typhus and Leptospira infections require 10‐day doxycycline and 7‐day penicillin respectively, both with 6‐month anticoagulation (Das et al. 2021; Turhan et al. 2006).

Viral etiologies such as varicella‐zoster virus demand acute intervention with acyclovir, seizure prophylaxis (e.g., phenytoin), and short‐term heparin, followed by 6 months of oral warfarin to reduce recurrence (Archana et al. 2022). HIV‐associated CVT requires 6–12 months of anticoagulation alongside highly active antiretroviral therapy (e.g., tenofovir, emtricitabine); coinfection with Cryptococcus neoformans adds prolonged amphotericin B and flucytosine for at least 2 weeks, combined with aggressive intracranial pressure control, though mortality remains high in this population (Equiza et al. 2020; Rakhra et al. 2021).

Parasitic causes, most notably hydatid cysts, rely on surgical resection as the cornerstone of care, followed by postoperative anti‐helminthic medications (e.g., albendazole) and anticoagulation, with optimal durations still undefined due to limited case reports (Namvar et al. 2023).

Infections occurring in the central face or paranasal sinuses can rapidly spread to the opposite side and further develop into septic cavernous sinus thrombosis(SCST) (Yuan et al. 2022). If not diagnosed and treated promptly, this can lead to serious complications or even death (Yuan et al. 2022). Otitogenic, odontogenic, and pharyngeal sources are relatively less common (Karakas et al. 2022). Otitogenic infections, especially when associated with cholesteatoma, are prone to causing sigmoid sinus thrombophlebitis. With the increase in antibiotic resistance, the incidence has begun to rise again (Yuan et al. 2022).

Infection‐related CVT management centers on infection control, with empiric antimicrobial regimens targeting predominant pathogens including Staphylococcus aureus, Gram‐positive/‐negative bacteria, and anaerobes through third‐generation cephalosporins combined with nafcillin and metronidazole (Khatri and Wasay 2016). Given the undefined optimal therapy duration and reported SCST recurrences 2–6 weeks post‐antibiotic cessation, extending therapy ≥2 weeks post‐symptom resolution is advised (Khatri and Wasay 2016).

Anticoagulation in infection‐related CVT remains controversial, with opponents arguing that septic thrombi may limit microbial dissemination despite lacking randomized trial support (Caranfa and Yoon 2021; José M. Ferro et al. 2004). While anticoagulation does not reduce mortality, early initiation (≤7 days post‐admission) correlates with lower neurological complication rates (31% vs. 59% for mild‐severe deficits) in survivors. Meningitis‐associated CVT warrants particular caution due to elevated intracranial hemorrhage risks (Zuurbier et al. 2016), accounting for the 3.3‐fold higher poor outcome probability observed in central nervous system infection cases (Ferro et al. 2004).

For otogenic thrombophlebitis, radical mastoidectomy may be considered when necessary, but the specific timing of the surgery remains uncertain. For patients with septic CVT, endovascular treatment may also be considered (Mascitelli et al. 2015), but whether surgery improves prognosis is still unknown.

#### 3.1.3 Mechanical Factors

Various mechanical triggers can also lead to CVT, including neurosurgical procedures, trauma to the skull, jugular vein catheterization, lumbar puncture and spinal anesthesia (Cha et al. 2022; Ma et al. 2024; Ren et al. 2020; Sturiale et al. 2023; Wu and Wang 2024; Zhang et al. 2024). The first three factors primarily cause vascular damage and endothelial dysfunction to promote blood coagulation, while lumbar puncture and spinal anesthesia are associated with excessive cerebrospinal fluid (CSF) withdrawal and the potential introduction of procoagulant substances during the procedure (Cha et al. 2022; Honig, Eliahou, Pikkel, and Leker 2016; Ren et al. 2020; Sturiale et al. 2023; Wu and Wang 2024; Zuurbier et al. 2015).

Postoperative CVT management remains contentious, with 78% of posterior fossa surgery‐related cases being asymptomatic (Sturiale et al. 2023). A retrospective analysis (n = 26) showed 12% developed neurological complications without anticoagulation (Orlev et al. 2020), while anticoagulation demonstrates increased intracranial hemorrhage risks (Shabo et al. 2023). Meta‐analyses show equivalent recanalization rates between treated/untreated groups, though limited by retrospective data predominance (Trevisi et al. 2024).

Traumatic CVT occurs in 44.3% of skull fractures involving venous sinuses, without 30‐day mortality difference versus non‐CVT trauma (17.9% vs. 18.5%) (Ma et al. 2024). Universal prophylactic anticoagulation in both groups precludes definitive therapeutic conclusions.

Catheter‐related CVT, primarily reported in chemotherapy patients, stems from catheter‐induced vascular injury per Virchow's triad (Larkey et al. 1993; Ren et al. 2020). Routine anticoagulation prevails despite unquantified risk‐benefit ratios and catheter removal uncertainties.

Lumbar puncture and spinal anesthesia represent uncommon etiologies of CVT, primarily associated with secondary intracranial hypotension (Honig et al. 2016). While CVT incidence in spontaneous intracranial hypotension (SIH) approximates 1%, its prevalence in secondary forms remains unestablished (Risi et al. 2024; Zhang, Wang, Zhang, He, and Hu 2018). Pathophysiologically, compensatory venous dilation per the Monro‐Kellie doctrine reduces cerebral venous flow velocities, promoting thrombosis (Risi et al. 2024). Management combines epidural blood patches (cornerstones of intracranial hypotension therapy) with anticoagulation, though the latter necessitates rigorous hemorrhage surveillance given elevated bleeding risks in hypotensive states (Risi et al. 2024; D. Zhang et al. 2018).

Unlike endogenous factors, CVT caused by mechanical injury does not advocate for aggressive anticoagulation, and it is even considered that the thrombus is a protective mechanism of the body. However, in current research, routine anticoagulation therapy for CVT patients is still the majority, except for asymptomatic CVT patients (Orlev et al. 2020; Shabo et al. 2023; Sturiale et al. 2023; Zhang et al. 2018).

### 3.2 Long‐Term Factors

Beyond transient mechanical triggers, long‐term systemic conditions such as autoimmune disorders, metabolic abnormalities, and malignancies constitute persistent risk profiles that require distinct therapeutic strategies.

#### 3.2.1 Autoimmune

Autoimmune diseases are important causes of CVT. The most common diseases leading to CVT include Behçet's syndrome (BS), systemic lupus erythematosus (SLE), antiphospholipid syndrome (APS), and Sjögren's syndrome (SS) (Zhang et al. 2021). Mixed connective tissue disease (Agrawal et al. 2024), inflammatory bowel disease (IBD) (De Cruz et al. 2008), Wegener's granulomatosis (Rostami et al. 2021), sarcoidosis (Selvi et al. 2009), thyroid disease (Fandler‐Höfler et al. 2022), and nephrotic syndrome (Balla, Hashi, Osman, Hassan, and Mutlu 2024) are less frequently associated. In recent years, paroxysmal nocturnal hemoglobinuria (PNH) (Ninan et al. 2023), immune thrombocytopenia (ITP) (Mahadevan et al. 2024), VEXAS syndrome (vacuoles, E1 enzyme, X‐linked, autoinflammatory, and somatic syndrome) (Zisapel et al. 2024), giant cell arteritis (Shafi and Kasner 2011), autoimmune hemolytic anemia (AIHA) (Kuroda, Itagane, and Kinjo 2021), and IgG4‐related disease (Ketabforoush et al. 2022) have also been reported as potential causes of CVT.

BS has been most extensively reported in relation to CVT. In the VENOST cohort, BS was identified as a causative factor for CVT in 108 out of 1144 patients (9.4%) (Uluduz et al. 2019). BS is considered a natural model of human inflammation‐induced thrombosis. Unlike traditional cardiovascular risk factors contributing to thrombosis, which primarily involve immune‐inflammatory responses, BS is mainly due to impaired immune‐inflammatory reactions. Specifically, excessive activation of neutrophils and neutrophil‐mediated damage mechanisms directly promote endothelial dysfunction, platelet activation, and thrombosis in BS (Bettiol et al. 2023).

Emerging evidence highlights the intracranial venous sinuses as immunologically active sites, equipped with full antigen‐presentation machinery and surveilled by adaptive immune cells (Engelhardt 2021). Flow cytometry analyses reveal B‐cell enrichment in murine sagittal/transverse sinuses, independent of bone marrow‐derived populations (Brioschi et al. 2021). Clinically, transverse sinuses represent the predominant thrombosis locus (48.2%), followed by superior sagittal sinuses (31.7%) in multicenter cohorts (Uluduz et al. 2019). These findings collectively implicate synergistic anatomical and immunological mechanisms in CVT pathogenesis.

Anticoagulant therapy in BS‐associated CVT lacks robust evidence for safety/efficacy despite 3‐year Western clinical application. Thrombosis in BS is primarily attributed to vasculitis‐driven inflammation, making anti‐inflammatory regimens often sufficient (Zhang et al. 2021). For APS‐related CVT, lifelong warfarin remains standard despite limited evidence, reflecting high recurrence risks. Autoimmune‐associated CVT generally exhibits favorable neurological outcomes when promptly treated, though delays incur permanent deficits (Aguiar de Sousa, Mestre, and Ferro 2011). Optimal anticoagulation strategies and duration in autoimmune CVT require further validation through controlled studies.

Emerging evidence links COVID‐19 vaccination to CVT and atypical thrombotic events, designated vaccine‐induced immune thrombotic thrombocytopenia (VITT) when platelet factor 4 (PF4) antibodies are detected (Joy et al. 2023; Sánchez Van Kammen et al. 2021). VITT clinically parallels heparin‐induced thrombocytopenia (HIT), with acute mortality reaching 40% during initial hospitalization, though follow‐up demonstrates low recurrence and bleeding risks (van de Munckhof et al. 2022). Diagnostic confirmation requires PF4 antibody ELISA in post‐vaccination CVT with thrombocytopenia (Gattringer et al. 2022). Management mandates heparin avoidance (including catheter flushes) due to cross‐reactivity risks. First‐line therapy combines intravenous immunoglobulin (IVIG) to neutralize PF4 antibodies with non‐heparin anticoagulants—fondaparinux, argatroban, or direct oral anticoagulants (Ropper and Klein 2021; Saposnik et al. 2024).

#### 3.2.2 Metabolic

Metabolic disorders are also significant pro‐coagulant factors that can affect the occurrence and development of CVT through various ways. Elevated plasma factor VIII (FVIII) levels are also considered an independent risk factor for CVT. In a case‐control study by Anadure, elevated FVIII (> 170 IU/dL) was associated with an over 18‐fold increased risk of non‐puerperal CVT. Additionally, patients with non‐O blood type generally have higher average FVIII levels (Anadure et al. 2014). Vecht also found in a case‐control study that elevated FVIII (> 150 IU/dL) was associated with a 15‐fold increased risk of CVT. When stratified by gender, it was observed that the correlation between elevated FVIII (> 150 IU/dL) and CVT risk was stronger in males compared to females (22.8‐fold vs. 14.7‐fold) (Vecht et al. 2018). A recent case‐control study also found that patients with non‐O blood type have approximately three times higher risk of CVT (Ken‐Dror et al. 2024). Therefore, in patients with elevated FVIII levels, especially males and those with non‐O blood type, vigilance for the occurrence of CVT is warranted. Aside from FVIII, vWF shows the strongest correlation with VTE (Rietveld et al. 2019). A case‐control study involving 25 CVT patients and 64 age‐ and gender‐matched controls, found a significant risk of CVT with vWF levels > 168% (Bugnicourt et al. 2006).

Anemia is considered a risk factor for CVT and a predictor of poor prognosis. A study involving 152 CVT patients and 2915 controls found that anemic patients had a 5.3‐fold increased risk of CVT, with a 9.9‐fold increased risk in males and a 3.6‐fold increased risk in females (Coutinho, Zuurbier, et al. 2015), while the reason is unknown. A retrospective study found that in CVT patients, anemia correlates with poor prognosis, particularly in cases of microcytic anemia. Anemia increased the risk of mRS ≥3 by 3.6‐fold (severe anemia, 8.8‐fold; microcytic anemia, 4.6‐fold) and mortality by 5.5‐fold (severe anemia, 9.8‐fold; microcytic anemia, 9.7‐fold) in CVT patients.(Liu et al. 2018) Another retrospective study involving 952 CVT patients found that anemia increased the risk of poor prognosis by 2.3‐fold (Silvis et al. 2020). Moreover, for patients with both anemia and cancer, the risk was further increased by 2.8‐fold compared to those with anemia alone, suggesting a potential interaction between anemia and cancer in influencing prognosis (Silvis et al. 2020). However, there is insufficient research to determine whether supplementing hemoglobin could improve the prognosis of CVT patients affected by anemia.

In addition to above factors, hypereosinophilia is occasionally reported in association with CVT, caused by allergic reactions, parasitic infections, autoimmune diseases, specific hematological disorders, or malignancies (Song et al. 2021). The prognosis of CVT linked to hypereosinophilia varies and largely depends on early detection and intervention (Song et al. 2021). Treatment typically involves corticosteroids to reduce eosinophil counts and anticoagulants to manage the condition (Song et al. 2021).

#### 3.2.3 Tumor

The incidence of tumors is increasing year by year and malignancies are present in 8.9% of CVT cases (hematological: 3.9%; solid tumors: 5.0%) (Silvis et al. 2018). Tumor‐derived procoagulants (tissue factor, oncofetal antigens) and iatrogenic factors drive thrombosis risk—L‐asparaginase in acute lymphoblastic leukemia (ALL) elevates blood viscosity via asparagine depletion, while steroids activate clotting mechanisms (De Stefano et al. 2015; Ranta et al. 2015). Pediatric ALL patients face additional risks: triglyceride levels > 615 mg/dL and increased body surface area (El‐Khoury et al. 2021). CVT incidence in ALL ranges 2–10.5%, with 10% mortality directly/indirectly attributable to thrombosis (Ghanem et al. 2017; Ranta et al. 2015). Therapeutic synergy between L‐asparaginase and dexamethasone necessitates vigilant thromboprophylaxis in this population (Alet et al. 2020; Ranta et al. 2015).

Myeloproliferative neoplasms (MPN), including essential thrombocythemia, polycythemia vera, and primary myelofibrosis, can also lead to CVT, of which the prognosis is generally good (Gangat et al. 2021). In a retrospective study of 74 CVT patients with MPN, with a median follow‐up of 5.1 years, the CVT recurrence rate was 4%, extracranial venous thrombosis rate was 9%, and arterial thrombosis rate was 11%, with no deaths attributable to CVT (Gangat et al. 2021).

According to data from VENOST study (a multicenter study of CVT), malignancy is associated with a 5.2‐fold to 9.9‐fold increase of CVT, and the malignancy increases the probability of poor outcomes (mRS ≥3) by 6.6‐fold in univariate analysis, and 3.3‐fold in multivariate analysis (Duman et al. 2017). A retrospective study in Finland found that 2.3% of CVT patients without cancer may be diagnosed with new cancer within 1 year, particularly in patients over 50 years old (Sipilä et al. 2022). A study in Denmark indicated that the risk of diagnosing new cancer within 3 months after CVT diagnosis increases by approximately 7‐fold (Sipilä et al. 2022). Therefore, CVT may be the precursor of tumor, and considering cancer screening is suggested for CVT patients over 50 years old without apparent causes.

Malignancy is independently associated with increased mortality, recurrence rates, and poor prognosis in CVT patients. According to International Study on Cerebral Vein and Dural Sinus Thrombosis (ISCVT) data, the presence of malignancy increases mortality by 8.33‐fold, (Ferro et al. 2004) which is not only due to its primary disease but also by increasing the occurrence of intracranial herniation. A single‐center prospective study found that the presence of malignancy increases the risk of intracranial herniation by 5.49‐fold (Dinç et al. 2023). A prospective observational study identified that the presence of cancer or malignant hematologic disease increased the recurrence rate of CVT by 3.2‐fold (Palazzo et al. 2017).

Although cancer is a risk factor, CVT incidence is lower compared to other thrombotic sites (Lyman et al. 2021). ESO guidelines advise against routine CVT screening in all cancer patients (Ferro et al. 2017). However, for cancer patients presenting with headache, nausea, transient visual impairment, papilledema, diplopia, seizures, or focal neurologic deficits, CTV/MRV is recommended for diagnostic evaluation.

Currently, research on anticoagulation in CVT patients with cancer remains limited. A recent systematic review and meta‐analysis including 17 randomized controlled trials involving 6623 patients with active cancer found that apixaban has favorable efficacy and safety profiles in preventing VTE recurrence, particularly in solid malignancies (Fujisaki et al. 2024). Given these findings, apixaban may be a reasonable option for patients with active cancer and CVT. However, further studies are needed to define its role in the management of CVT in this population.

### 3.3 Permanent Factors

#### 3.3.1 Hereditary Thrombophilia Condition

In contrast to acquired risks like malignancy, inherited thrombophilia embodies non‐modifiable, lifelong predispositions to CVT, necessitating genetic evaluation and personalized anticoagulation plans.

Protein C (PC), protein S (PS), and antithrombin (formerly known as antithrombin III) are the most important natural anticoagulants (Lipe and Ornstein 2011). Protein S or protein C deficiency, activated protein C resistance, and antithrombin deficiency are considered risk factors for CVT. In CVT patients, approximately 5% have protein S or protein C deficiency, 1.5% have activated protein C resistance, and 0.5% have antithrombin deficiency (Duman et al. 2017).

Factor V Leiden (FVL) and prothrombin G20210A mutations are established genetic risk factors for CVT, mediated through activated protein C resistance and elevated prothrombin activity respectively (Li et al. 2018; Marjot et al. 2011). These thrombophilic mutations demonstrate synergistic effects with environmental triggers—particularly oral contraceptive use, which amplifies thrombotic risks in mutation carriers via estrogen‐mediated coagulation pathway activation (Dentali et al. 2006).

The methylenetetrahydrofolate reductase (MTHFR) C677T polymorphism reduces enzymatic activity, elevating plasma homocysteine levels and conferring prothrombotic risk (Gouveia and Canhão 2010; Martinelli, Battaglioli, Pedotti, Cattaneo, and Mannucci 2003). While hyperhomocysteinemia is implicated in CVT pathogenesis, conclusive evidence establishing the 677TT genotype's role remains lacking, necessitating further mechanistic investigation.

Recent research has also identified F9 gene duplication leading to elevated factor IX activity levels as a new thrombotic risk factor for CVT (Tan et al. 2023). Additionally, mutations in the Retinoic Acid‐Induced 1 (RAI1) gene, which cause Smith‐Magenis syndrome, may also increase the risk of CVT (Zahoor et al. 2023).

Warfarin, as a vitamin K antagonist, inhibits the body's production of protein C and protein S. Patients with deficiencies in protein C or protein S are not recommended to undergo warfarin therapy without concurrently receiving another anticoagulant. Additionally, oral contraceptives can reduce protein S levels by approximately 20% (Alving and Comp 1992). Testing should be performed at least several weeks after acute thrombotic events or cessation of warfarin or heparin for 3–6 weeks to avoid false‐positive results.

According to the American Society of Hematology 2023 guidelines on venous thromboembolism management and the scientific statement from the American Heart Association, thrombophilia testing is recommended for patients who have completed initial anticoagulation therapy (usually 3–6 months) (Middeldorp et al. 2023; Saposnik et al. 2024). This testing should be conducted 3–6 weeks after discontinuing anticoagulants to avoid false‐positive results due to acute thrombotic events or interference from anticoagulant medications (Middeldorp et al. 2023). When the test indicates a hypercoagulable state associated with a high risk of recurrence (such as deficiencies in antithrombin, protein C/S, or positivity for antiphospholipid antibodies), indefinite anticoagulation therapy should be considered, as the annual recurrence risk can exceed 10% (Prandoni et al. 2012).

However, indefinite anticoagulation can significantly increase the risk of bleeding (with an annual major bleeding rate of approximately 2%) and impose a long‐term economic burden on younger patients (van de Munckhof et al. 2025). Therefore, for patients with transient risk factors (such as surgery, trauma, or hormone therapy) that have been completely resolved, discontinuation of anticoagulation after 12 months is a reasonable option if the following conditions are met: ① normalization of D‐dimer levels; ② no residual thrombi on imaging; ③ absence of other high‐risk factors (such as obesity or active cancer) (Pinède 2001; Prandoni et al. 2012).

For patients with CVT who have the aforementioned high‐risk thrombophilia, indefinite treatment is still recommended even after 12 months of anticoagulation (Pinède 2001). For those with intermediate‐risk thrombophilia (isolated factor V Leiden or prothrombin G20210A mutation), the current evidence shows an annual recurrence risk of 3–5%, which is similar to that of typical idiopathic VTE patients (Pinède 2001). Therefore, whether to extend treatment beyond 12 months should be based on dynamic assessments (such as D‐dimer levels and residual thrombi) and individualized risk‐benefit analyses (Middeldorp et al. 2023). More prospective studies are still needed to clarify this issue.

Complementing these established categories, diverse exogenous exposures ‐including substance use, environmental triggers, and rare comorbidities—further expand the etiological spectrum of CVT. Clinicians should still try to intervene in controllable factors during clinical practice in order to mitigate the progression of thrombosis and severe complications.

### 3.4 Other Factors

Other risk factors include active smoking (Cohen et al. 2024), excessive alcohol consumption (Patil et al. 2024), dehydration (Patil et al. 2024), abuse of androgens (Lippi and Banfi 2011), nitrious oxide (N_2_O) (Banjongjit et al. 2023), sildenafil (Karti, Karti, Aktert, Gokcay, and Celebisoy 2017), Tetralogy of Fallot (Geva et al. 1990), dural fistulas (Wan et al. 2024), venous anomalies (Chang, Rebchuk, Teal, Honey, and Field 2022), wasp stings (J. Zhang et al. 2024), snakebites (Ghosh et al. 2022) etc. The mechanisms are various and complexed.

## 4 Therapeutic Management of CVT

### 4.1 Anticoagulation Therapy

Anticoagulant therapy is the cornerstone of the management of CVT, which can effectively reduce the risk of venous VTE recurrence by inhibiting the progression of thrombosis and promoting vascular recanalization. The commonly used anticoagulation regimens include parenteral anticoagulation therapy, vitamin K antagonists, and direct oral anticoagulants.

#### 4.1.1 Parenteral Anticoagulation Therapy

The American Heart Association/American Stroke Association (AHA/ASA) and the European Stroke Organization (ESO) endorse the use of unfractionated heparin (UFH) or low molecular weight heparinoids (LMWHs) during the acute phase of CVT (J. M. Ferro et al. 2017; Saposnik et al. 2024). A meta‐analysis involving 14 studies with 1,135 patients has confirmed that anticoagulant treatment with heparin is safe and effective for managing CVT, including cases with concurrent hemorrhagic stroke caused by CVT (Xu et al. 2018).

Additionally, for pregnant women with a previous history of CVT, the ESO guidelines advocate for prophylaxis with subcutaneous LMWH during pregnancy and the puerperium, provided there are no contraindications for prophylaxis or indications for therapeutic‐dose anticoagulation (Ferro et al. 2017).

#### 4.1.2 Vitamin K Antagonist

After initiating anticoagulation with LMWH or UFH, one must bridge to oral anticoagulation with a vitamin K antagonist (VKA) to promote recanalization and prevent CVT recurrence or other venous thromboembolic events, with a target international normalized ratio of 2 to 3 for 3 to 6 months in patients with provoked CVT, 6 to 12 months in those with unprovoked CVT, and indefinite anticoagulation in patients with recurrent CVT, VTE after CVT, or those with severe thrombophilia experiencing their first CVT event (Saposnik et al. 2024).

#### 4.1.3 Direct Oral Anticoagulants

Accumulating evidence supports direct oral anticoagulants (DOACs) as therapeutic alternatives to VKAs for CVT. Regarding recurrent thrombotic events, clinical trial data consistently indicate low absolute rates in both treatment groups. Smaller trials reported few recurrent events overall. RE‐SPECT CVT and EINSTEIN‐Jr documented only 0–1 recurrent events per study arm (Connor et al. 2020; Ferro et al. 2019). Three trials — SECRET (2/26 [7.7%] vs. 0/29 [0%]), CHOICE‐CVT (8/44 [18.2%] vs. 3/45 [6.7%]), and DOAC‐CVT (9/130 [6.9%] vs. 3/132 [2.3%]) —observed numerically higher recurrence rates with DOACs compared to VKAs. However, none of these differences reached statistical significance (Field et al. 2023; Ma et al. 2024; van de Munckhof et al. 2025). Similarly the large, prospective ACTION‐CVT trial (*n* = 845) found a lower annualized recurrence rate with DOACs (5.26 per 100 patient‐years) compared to VKAs (5.87 per 100 patient‐years), though this difference was also not statistically significant (hazard ratio [HR] 0.94, 95% confidence interval [CI] 0.51–1.73; Yaghi et al. 2022).

Major bleeding events were relatively uncommon across studies. However, findings regarding bleeding risk exhibited some inconsistency: The SECRET trial reported intracranial hemorrhage in 2 of 26 (7.7%) DOAC recipients compared with none in the VKA group. The ACTION‐CVT trial demonstrated major bleeding rates of 2.44 versus 4.70 events per 100 patient‐years for DOACs and VKAs, respectively (HR 0.35, 95% CI 0.15–0.82; *p* = 0.06). Conversely, the DOAC‐CVT trial reported symptomatic major bleeding in 1% of DOAC recipients versus 2% in the VKA group, and clinically relevant non‐major bleeding in 2% versus 1%. Collectively, these data indicate that DOACs and VKAs are associated with broadly comparable rates of recurrent venous thrombosis and major bleeding in patients with CVT, although subtle numerical differences exist that warrant consideration in individual patient management decisions (Field et al. 2023; van de Munckhof et al. 2025; Yaghi et al. 2022).

DOACs require population‐specific considerations: Dabigatran (150 mg bid) demonstrates comparable venous recanalization and lower hemorrhage risk versus warfarin in uncomplicated acute CVT (H. Ma et al. 2024). Rivaroxaban is preferred for short‐term (3–6 months) cancer‐associated CVT due to superior VTE prevention over LMWH, while also showing pediatric efficacy with controlled bleeding (Field et al. 2023; Lyman et al. 2021). Apixaban optimizes long‐term (≥6 months) cancer therapy with equivalent VTE prevention and minimized bleeding (2.5 mg bid dose maintains efficacy), particularly suitable for elderly and high‐bleeding‐risk patients (Fujisaki et al. 2024; Mahé et al. 2025). DOACs remain contraindicated in mechanical heart valves, triple‐positive antiphospholipid syndrome, and lactation (excluding edoxaban) (Monagle et al. 2012; van de Munckhof et al. 2025).

#### 4.1.4 Anticoagulation Duration

According to the American Heart Association/American Stroke Association (AHA/ASA) and European Stroke Organization (ESO) guidelines, the duration of anticoagulation for CVT needs to be stratified based on the individual patient's condition: 3–6 months of anticoagulation is recommended for patients with secondary CVT; 6–12 months is recommended for patients with a first episode without a clear cause; and those with a history of recurrent CVT, VTE, or a predisposition to severe thrombosis require long‐term or even Long‐term or even lifelong anticoagulation is recommended for patients with recurrent CVT, VTE, or a predisposition to severe thrombosis (Ferro et al. 2017; Saposnik et al. 2024).

With regard to children and the evidence base for short‐course treatment, several studies have shown that individualized adjustments can shorten the treatment period. In pediatric patients with short‐term risk factors such as infection or trauma, the duration of anticoagulation can be shortened to 6 weeks to 3 months (Monagle et al. 2012). A retrospective study of 53 children in Portugal, with a median age of 5 years, demonstrated the efficacy of anticoagulation for a median of 6 months in preventing recurrence during the 3‐year follow‐up period (Rodrigues et al. 2022). The Kids‐DOTT randomized controlled study (417 patients aged 4 months to 20 years) further demonstrated that 6 weeks of anticoagulation had a non‐inferior risk of thrombotic recurrence to a 3‐month course at 1 year and subsequent 2‐year follow‐up (Goldenberg et al. 2022).

For patients with long‐term risk factors (autoimmune disease, tumors etc.), the guidelines recommend extending anticoagulation to 6–12 months, and for those at high risk of recurrence (e.g., antiphospholipid syndromes, inherited thrombophilic disorders), the American Society of Haematology 2023 guidelines explicitly recommend permanent anticoagulation (Middeldorp et al. 2023).

### 4.2 Endovascular Therapy

We summarize 20 endovascular studies published between 2007 and 2021 (Table 1). Across these reports, intracranial hemorrhage occurred in 0–33% of cases, mortality in 0–19%, and favorable functional outcome (mRS 0–1) in 26–100%. Most notably, the rate of “complete recanalization” declined from 62–100% in early series to 29–81% in more recent ones, reflecting the adoption of stricter angiographic definitions that require the absence of residual thrombus rather than simple restoration of flow.

**TABLE 1:** Endovascular treatments: a summary of patient criteria, procedures, outcomes and complications across studies.

Selection criteria	Procedure types	Outcomes	Complications and incidence rate	Study
ICH, a hematoma, or edema	EMT ± (CDT + AP)	mRS 0–1: 9/15	Hemorrhage: 6/15	(Tsai et al. 2007)
Altered mental status, coma, straight sinus thrombosis, or large space‐occupying lesions	CDT ± EMT	mRS 0–2: 12/20	Hemorrhage: 5/20 Death: 6/20	(Stam et al. 2008)
Contraindication to SAC, SAC failure	CDT ± (EMT + AP)	mRS 0–1: 11/16 Recanalization: partial 10/16 (62.5%), complete 1/16 (6.3%)	Death: 3/16	(La Barge et al. 2009)
CVT patients	EMT	mRS 0–1: 7/9 mRS 0–2: 9/9 Recanalization: complete 13/13 (100%)	None	(Dashti et al. 2013)
SAC failure	EMT + CDT	mRS 0–1: 34/52 mRS 0–2: 40/52 Recanalization: Complete 45/52 (87%), Partial 3/52 (6%), None 4/52 (8%)	Death: 6/52	(Li et al. 2013)
Extensive involvement of sinuses with altered mental status/coma; Deterioration of symptoms despite being on anticoagulation, large space‐occupying lesions, such as edema or (hemorrhagic) infarcts	EMT ± CDT; CDT	mRS 0–1: 33/53	Hemorrhage: 12/63 Death: 11/63	(Siddiqui et al. 2014)
CVT patients	EMT + AP	mRS 0–1: 26/26 Recanalization: complete 26/26 (100%)	None	(Shui et al. 2014)
CVT patients	EMT ± CDT	mRS 0–2: 5/11	Death: 3/11	(Mokin et al. 2015)
CVT patients	EMT	NIHSS improvement: 23/23	None	(Ma et al. 2016)
ICH, infarction	EMT ± CDT	mRS 0–1: 16/29 mRS 0–2: 21/29	Hemorrhage: 9/29	(Nyberg et al. 2017)
Cerebral edema, herniation, or hemorrhagic infarction; neurological deterioration; re‐thrombosis, persistent occlusion, or clot propagation; extensive clot burden; persistent headache despite anticoagulation	EMT ± CDT ± AP	mRS 0–1: 31/73 Recanalization: complete 61%, partial 30%, none 9%	Hemorrhage: 7/73	(Salottolo et al. 2017)
Neurological deterioration, somnolence or coma, or venous cerebral infarction with hemorrhagic transformation or ICH following anticoagulation	EMT + CDT + AP	mRS 0–1: 6/14 Recanalization: complete 4/10, improved 6/10	Death: 1/14	(Qureshi et al. 2018)
Altered mental status or coma, deep vein involvement, ICH, coma, cerebral venous infarction, seizures, cerebral edema/midline shift, respiratory failure	EMT	mRS 0–2: 12/13 Recanalization: technical success 86% (complete 29%, partial 57%)	Death: 1/13	(Styczen et al. 2019)
CVT patients	EMT ± CDT; CDT	Death: 198/1248	NA	(Siddiqui et al. 2019)
Deterioration of initial clinical status, ICH, seizures	EMT	mRS 0–3: 9/16 Recanalization: 100% good (complete + partial)	None	(Dandapat et al. 2019)
Deterioration of consciousness, aggravation of neurological deficits and/or exacerbation of seizures after anticoagulation	EMT; CDT	mRS 0–1: 26/30 Recanalization: complete 42.9%, partial 50%, failed 7.1%	Hemorrhage: 2/30 Death: 2/30	(Liao et al. 2020)
Clinical deterioration and/or consciousness impairment post‐anticoagulation	EMT ± CDT; CDT	mRS 0–2: 18/28 Recanalization: complete 54%, partial 39%, none 7%	Hemorrhage: 9/28 Death: 5/28	(Andersen et al. 2021)
Altered mental status, coma (GCS < 9), ICH, or deep brain venous system thrombosis despite anticoagulation	EMT ± CDT	mRS 0–1: 22/33 Recanalization: complete 79%	Hemorrhage: 1/33 Death: 4/33	(Coutinho et al. 2020)
ICH	CDT ± EMT	mRS 0–2: 49/56 Recanalization: complete 67.8%, partial 26.9%	Hemorrhage: 5/56	(Guo et al. 2020)
Rapid neurologic decline post‐anticoagulation, somnolence/coma, hemorrhagic venous infarction, ICH, herniation from large hematoma/diffuse edema	EMT + CDT + AP	mRS 0–1: 21/23 Recanalization: complete 81%, partial 19%	None	(Yang et al. 2021)

Abbreviations: AP, angioplasty; CDT, catheter‐directed thrombolysis; EMT, endovascular mechanical thrombectomy; ICH, intracerebral hemorrhage; NA, not available. AP, angioplasty; CDT, catheter‐directed thrombolysis; EMT, endovascular mechanical thrombectomy; ICH, intracerebral hemorrhage; NA, not available.

Building on these observations, the following sections dissect the three principal endovascular modalities—mechanical thrombectomy, catheter‐directed thrombolysis, and angioplasty/stenting—detailing their respective mechanisms, technical nuances, and clinical performance profiles.

#### 4.2.1 Endovascular Mechanical Thrombectomy

Endovascular mechanical thrombectomy (EMT) encompasses direct aspiration thrombectomy and stent‐retriever thrombectomy (Kan et al. 2024). Direct aspiration thrombectomy involves crushing the thrombus with a guide wire and then applying negative pressure vacuum to aspirate the thrombus fragments (Kan et al. 2024). The commonly used aspiration systems include the AngioJet System and the Penumbra System (Siddiqui et al. 2015). A retrospective study indicates that compared to the Penumbra System, the AngioJet System is associated with a lower likelihood of good prognosis and poorer recanalization rates, with only about 55% of patients treated with the AngioJet System achieving complete recanalization (Siddiqui et al. 2015). Stent‐retriever thrombectomy involves navigating a catheter to the thrombus location, deploying a stent that expands and serves as an anchor, engaging and embedding itself within the thrombus, and then retracting the stent along with the thrombus using a sheath or catheter's suction. The use of Solitaire stents and Trevo devices in CVT has been reported (Byer, Madarang, and Abraham 2023; Mascitelli et al. 2015). However, with the technologies mentioned, EMT is not effective in removing non‐acute thrombi (Kan et al. 2024).

#### 4.2.2 Catheter‐Directed Thrombolysis

Catheter‐directed thrombolysis involves injecting thrombolytic drugs directly into the thrombus within the venous sinus through a catheter, but isolated catheter‐directed thrombolysis for CVT is now very rare (Cabral de Andrade et al. 2017). Urokinase, streptokinase, and rt‐PA have been used as thrombolytic agents for the treatment of CVT (Raza et al. 2020). Catheter‐directed thrombolysis is often combined with EMT, including intraoperative adjuvant periodic thrombolysis and postoperative continuous thrombolysis (Siddiqui et al. 2015). Intraoperative adjuvant periodic thrombolysis aids in thrombus dissolution to facilitate mechanical manipulation (Siddiqui et al. 2015). Postoperative continuous thrombolysis can clear residual thrombus after surgery, especially those that are difficult to completely remove mechanically, while also preventing postoperative vessel restenosis or re‐occlusion (Siddiqui et al. 2015). However, even with intrasinus thrombolysis, effectively clearing non‐acute thrombi remains a challenge (Kan et al. 2024).

#### 4.2.3 Angioplasty

Angioplasty includes stent implantation and balloon angioplasty, typically used as an adjunctive treatment to EMT (Kan et al. 2024). Balloon angioplasty is used to open narrowed vascular channels to allow for the smooth passage of thrombectomy devices to the thrombus location. It can be used in conjunction with EMT and/or intrasinus thrombolysis (Cabral de Andrade et al. 2017). Stent implantation is usually performed in conjunction with stent‐retriever thrombectomy. Due to the need for long‐term prevention of in‐stent restenosis with prolonged stent placement, long‐term anticoagulation and antiplatelet therapy are required, which increases the risk of bleeding for the patient, and thus stents are typically not implanted in CVT patients (Raza et al. 2020).

## 5 Current Issues in CVT Management

### 5.1 Incomplete Thrombus Clearance in Anticoagulation Therapy

Despite the varying risk factors that lead to CVT, the anticoagulation regimen typically involves treatment for 3–12 months or indefinite anticoagulation therapy. Long‐term anticoagulation therapy increases the risk of bleeding events in patients, with the incidence of major bleeding ranging from 2.44 to 4.70 per 100 patient‐years and the probability of intracranial hemorrhage being approximately 1.52 to 3.51 per 100 patient‐years (Yaghi et al. 2022). Despite long‐term anticoagulation, some patients still experience recurrent VTE, with an incidence of about 5.26 to 5.87 per 100 patient‐years (Yaghi et al. 2022). Additionally, about 15% of patients have persistent vessel occlusion, with residual thrombi remaining in their blood vessels after anticoagulation therapy (Yaghi et al. 2022).

### 5.2 Hard to Assess Non Neurological Deficit Symptoms

Like cerebral infarction caused by arterial thrombosis, mRS is used to evaluate the outcome and prognosis of CVT. Timely diagnosis and early initiation of anticoagulation therapy appear to yield favorable outcomes (mRS 0–1) in approximately 68–80% of patients (Coutinho et al. 2020; Dentali et al. 2012; Jedi et al. 2022; Le et al. 2024; Siddiqui et al. 2019; Zhou et al. 2024). However, the mRS score can only partially reflect the disabilities of the CVT patients. Some patients have non‐neurological deficits such as headache and cognitive impairment, which cannot be evaluated using the mRS score, leading to an underestimation of the adverse prognosis caused by CVT. It is reported that non‐neurological deficits affect around 70% of patients, including chronic headaches (20–59%), fatigue (30–37%), neurocognitive deficits (41–75%), anxiety (28–31%), and depression (16–30%), as shown in Table 2 (Bossoni et al. 2022; Coutinho et al. 2024; Dias, João Pinto, Maia, Albuquerque, and Carvalho 2024; Hiltunen et al. 2016, Hiltunen et al. 2016; Lindgren et al. 2018; Patwardhan et al. 2024).

**TABLE 2:** Long‐term non‐mRS sequelae after CVT: Patient‐reported outcomes and pooled incidence rates.

Sequelae type	Pooled incidence	Available quantitative tools	References
Chronic headache	20–59%	HIT‐6	(Bossoni et al. 2022; Dias et al. 2024; Hiltunen et al. 2016; Koopman et al. 2009; Lindgren et al. 2018)
Fatigue	30–37%	FSS	(Hiltunen et al. 2016; Koopman et al. 2009; Lindgren et al. 2018)
Cognitive disorder	41–75%	TICS‐M	(Bossoni et al. 2022; Hiltunen et al. 2016; Koopman et al. 2009; Lindgren et al. 2018; Patwardhan et al. 2024)
Anxiety	28–31%	GAD‐7/HADS‐A	(Bossoni et al. 2022; Lindgren et al. 2018)
Depression	16–30%	PHQ‐9/HADS‐D/CES‐D/BDI	(Bossoni et al. 2022; Dias et al. 2024; Hiltunen et al. 2016; Koopman et al. 2009; Lindgren et al. 2018; Patwardhan et al. 2024)

Abbreviations: BDI, beck depression inventory; CES‐D: center for epidemiological studies depression scale; FSS, fatigue severity scale; GAD‐7, generalized anxiety disorder‐7; HADS‐A, hospital anxiety & depression scale–anxiety; HADS‐D, hospital anxiety & depression scale–depression; HIT‐6, headache impact test‐6;PHQ‐9, patient health questionnaire‐9; TICS‐M, modified telephone interview for cognitive status. BDI, beck depression inventory; CES‐D, center for epidemiological studies depression scale; FSS, fatigue severity scale; GAD‐7, generalized anxiety disorder‐7; HADS‐A, hospital anxiety & depression scale–anxiety; HADS‐D: hospital anxiety & depression scale–depression; HIT‐6, headache impact test‐6;PHQ‐9, patient health questionnaire‐9; TICS‐M, modified telephone interview for cognitive status.

A cross‐sectional study reported that among patients followed up between 6 months and 5 years post‐CVT diagnosis (*n* = 100; 82% female), 59% experienced post‐CVT headache (PCH), with 52.6% presenting with tension‐type headache and 27.1% with migraine (Bossoni et al. 2022). A retrospective study found that 45.8% of patients reported PCH at 3‐month follow‐up, with 73.3% experiencing new‐onset headache (Dias et al. 2024). Furthermore, research has highlighted the prevalence of long‐term sequelae such as attention deficits, fatigue, and depression, which can occur even in patients without cognitive impairment. For instance, an investigation with a median follow‐up of 63 months found that 75% of patients reported attention deficits, 43% experienced headaches, 30% suffered from depression, and 30% felt fatigued (Koopman et al. 2009). A Canadian study demonstrated that among surviving patients with acute complications, 42.10% exhibited cognitive dysfunction and 16.66% showed signs of depression (Patwardhan et al. 2024). Bruijn et al. conducted an 18.5‐month follow‐up on 47 CVT patients and found that 40% of survivors who did not regain their previous work capacity had mild or moderate disability (mRS ≤2), with nearly half experiencing cognitive impairment (Liu et al. 2023).

Such long‐term patient‐centered outcomes underscore the lasting impact of CVT and emphasize the need for extended follow‐up and comprehensive management strategies. In addition to the mRS, a comprehensive assessment is essential for evaluating the overall condition of CVT patients and guiding long‐term follow‐up. Headache can be assessed with the 6‐item Headache Impact Test (HIT‐6) (Koopman et al. 2009). Fatigue is quantified using the 9‐item fatigue severity scale (FSS) (Koopman et al. 2009). Depression can be evaluated via the patient health questionnaire‐9 (PHQ‐9), the 20‐item Center for Epidemiological Studies depression scale (CES‐D) and the hospital anxiety & depression scale–depression (HADS‐D), and the Beck depression inventory (BDI) (Bossoni et al. 2022; Hiltunen et al. 2016; Koopman et al. 2009; Lindgren et al. 2018; Patwardhan et al. 2024). Anxiety is measured by the generalized anxiety disorder 7 (GAD‐7) scale and the hospital anxiety & depression scale–anxiety (HADS‐A) (Koopman et al. 2009; Lindgren et al. 2018). Cognitive impairment is assessed using the mini‐mental state examination (MMSE) and the modified telephonic interview for cognitive status (TICS‐M) (Table 2) (Bossoni et al. 2022; Patwardhan et al. 2024). These tools would enhance the availability to comprehensively monitor and manage the long‐term effects of CVT on patients.

### 5.3 Lack of Uniform Evaluation Criteria for Recanalization and Thrombus Burden

Early classifications for assessing recanalization in CVT relied on the Qureshi score (Qureshi 2010). This three‐tier system defined: Grade I (persistent occlusion of all initially thrombosed segments, with possible collateral improvement); Grade II (residual occlusion in ≥1 segment with recanalization elsewhere); and Grade III (no persistent occlusion). Unfortunately, Grade III did not differentiate between partial and complete recanalization. To address this limitation, de Sousa et al. introduced a refined four‐tier scale (0–3) (Aguiar de Sousa et al. 2020): Grade 0: Persistent occlusion of all segments (equivalent to Qureshi Grade I);Grade 1: Residual occlusion in ≥1 segment with recanalization elsewhere (Qureshi Grade II);Grade 2: Absence of persistent occlusion but incomplete recanalization in ≥1 segment; Grade 3: Complete recanalization of all segments. Notably, in the Qureshi score, Grade II is typically considered to represent partial recanalization. The de Sousa scale further refines this concept: both its Grade 1 (residual occlusion in ≥1 segment) and Grade 2 (incomplete recanalization in ≥1 segment without persistent occlusion) represent distinct manifestations of partial recanalization. Owing to its segment‐level granularity, the de Sousa scale has become the preferred framework for recanalization assessment in recent CVT studies (Field et al. 2023; van de Munckhof et al. 2025).

A second approach quantifies total thrombus burden with a “CVT score.” This method assigned one point to each occluded segment among 15 predefined sites (Afifi et al. 2020; Schuchardt et al. 2023) compare pre‐ and post‐treatment scores of overall recanalization. However, it cannot distinguish partial from complete reopening within individual segments.

More recently, Wang et al. (2023) proposed a refined “recanalization score” based on black‐blood MRI (Wang et al. 2023). Each segment is graded 0 (no thrombus), 1 (≤50% lumen occupied), 2 (> 50% but < 100%), or 3 (complete occlusion) (Wang et al. 2023). Dominant sinuses are further weighted for cross‐sectional area, yielding a 15‐site map. This 0–3 scale provides segment‐by‐segment detail, while its reliance on specialized black‐blood sequences currently limits widespread adoption (Wang et al. 2023).

The clinical implementation of the novel evaluation system faces significant challenges due to the technical limitations of black‐blood sequence imaging, which requires specialized equipment and technical expertise. Currently, this technology demonstrates limited availability and low utilization rates in most medical institutions, hindering widespread adoption of the new scoring system. To address these barriers, multicenter trials are recommended to validate its effectiveness and reliability through large‐scale clinical datasets. Concurrently, standardized training programs and knowledge‐sharing platforms should be established to enhance operational proficiency in black‐blood sequence imaging. Institutions with suboptimal technical readiness may temporarily maintain the conventional CVT scoring system to ensure clinical continuity, with phased transitions implemented upon achieving technical maturity. These strategies collectively aim to optimize the CVT assessment system through evidence‐based validation and progressive integration.

### 5.4 Safety and Effectiveness of EVT Remain to be Proven

EVT has been associated with higher rates of vascular recanalization, yet most evidence suggesting improved clinical outcomes in CVT patients post‐EVT is derived from small‐sample retrospective studies or case reports. Additionally, EVT carries potential risks such as intraoperative sinus rupture or tear, postoperative new‐onset or worsening intracranial hemorrhage ICH, and other major hemorrhagic complications (Tang et al. 2023). We outlines patient selection criteria, procedure types, clinical outcomes, and complication rates from multi‐center studies of the current application of EVT in Table 1.

The randomized controlled trial, TO‐ACT, which investigated the efficacy of EVT in high‐risk CVT patients, did not demonstrate statistically significant benefits and was prematurely terminated due to futility during interim analysis (Coutinho et al. 2020). The failure of this study may be attributed to several factors related to patient demographics, endovascular treatment devices, and outcome measures. First, the intervention group included older patients, a lower proportion of female patients, and a higher number of patients without headache symptoms—factors previously associated with poorer outcomes in vascular events (Coutinho et al. 2015; Duman et al. 2017). Additionally, the intervention group had a higher prevalence of prior VTE, a condition that has been linked to adverse outcomes in earlier research (Shu et al. 2022). The majority of patients in the study were treated using the AngioJet device, which retrospective studies have suggested may result in lower recanalization rates and a decreased likelihood of favorable outcomes (Siddiqui et al. 2015). It is noted that the proportion of patients achieving an mRS score of 0 was higher in the intervention group, though this difference did not reach statistical significance due to the small sample size (Coutinho et al. 2020). The distinction between mRS scores of 0 and 1, which reflects the presence of residual symptoms, is critical in evaluating the efficacy of treatment.

Previous studies have demonstrated that endovascular treatment can increase vascular recanalization rates (Naik et al. 2022). In this study, the intervention group demonstrated significantly higher rates of sinus recanalization compared to the standard treatment group (Coutinho et al. 2020). Therefore, endovascular treatment may play a crucial role in reducing the incidence of post‐stroke sequelae. Based on current evidence, it is recommended that patients with a high demand for quality of life, particularly those without risk factors such as edema, infarction, or hemorrhage, consider endovascular treatment. Future prospective studies are needed to investigate this issue more comprehensively.

### 5.5 Future Directions and Perspectives for Treatment

In the future direction and outlook of treatment, the optimization of thrombectomy therapy is a crucial link. At present, EVT, as a second‐line treatment for CVT, is only recommended for high‐risk patients with worsening symptoms (such as neurological deterioration, coma, intracranial hemorrhage etc.) after anticoagulant therapy. The TO‐ACT randomized controlled trial did not show a significant benefit of EVT, which largely attributed to patient selection bias and device limitations (Coutinho et al. 2020). Future thrombectomy therapy needs to combine the acute degree of thrombosis, thrombus burden and clinical deterioration speed, and screen the patients with the greatest benefit for intervention. Despite the current availability of various EVT methods, like mechanical thrombectomy, catheter‐directed thrombolysis and angioplasty, it is necessary to establish standards and accumulate more evidence‐based evidence for EVT. The strategy of stent thrombectomy combined with postoperative continuous thrombolysis seems be recommended. In this method, most of the thrombus is removed by stent thrombectomy during the operation, and low dose rt‐PA is injected into the residual thrombus through microcatheter after the operation, which can not only improve the recanalization rate, but also reduce the risk of intraoperative bleeding. In addition, special stent retrievers or biodegradable stents suitable for veins can be developed in the future to avoid in‐stent restenosis caused by long‐term indwelling, reduce the combined use of anticoagulant and antiplatelet drugs, and further improve the quality of life of patients.

The definition and evaluation of vascular recanalization require further refinement. While the de Sousa scale (0–3), now widely adopted as the preferred framework, offers significant advantages over earlier systems like the Qureshi score by clearly defining and differentiating states of partial recanalization (Grades 1 & 2), it remains a primarily qualitative assessment at the segment level. The CVT score quantifies overall thrombus burden but lacks segmental resolution to distinguish degrees of recanalization. The more granular black‐blood MRI recanalization score (0–3 per segment) proposed by Wang et al. represents a step towards quantification of the *degree* of reopening within each segment, directly addressing the limitation inherent in the de Sousa Grade 2 category (“incomplete recanalization”). However, its reliance on specialized sequences currently limits widespread clinical adoption  (Wang et al. 2023). Therefore, building upon the foundation of the de Sousa scale, future refinement of recanalization assessment should aim to establish quantitative thresholds within the “partial recanalization” spectrum. It seems feasible to refine the “partial recanalization” category (encompassing de Sousa Grades 1 & 2) by defining subgrades such as: “significant recanalization” (e.g., > 50–99% lumen patency) and “minor recanalization” (e.g., 1–50% patency), alongside “complete recanalization” (100% patency, de Sousa Grade 3) and “persistent occlusion” (0% patency, de Sousa Grade 0). Validation would require multimodal imaging cross‐correlation and potentially incorporating functional indicators like venous pressure monitoring or cerebral hemodynamics assessment for a more comprehensive and accurate picture.

In terms of outcome evaluation, it is necessary to break through the limitations of mRS and pay attention to the long‐term patient‐centered quality of life. At present, the outcome evaluation of CVT is mainly based on mRS, and about 68–80% of patients have a mRS score of 0–1 at 12 months, indicating a good prognosis. However, mRS can only reflect limb functions and cannot capture non‐disabling symptoms such as headache, fatigue, and cognitive impairment that affect the quality of life. In the future, mRS should be combined with other scales (such as headache impact test‐6, fatigue severity scale, FSS, PHQ‐9 etc.) to comprehensively obtain the long‐term impact on the quality of life of CVT patients (Koopman et al. 2009; McGeary et al. 2022; Negeri et al. 2021). In addition, the changes of headache, cognitive function, fatigue and other symptoms during the treatment should be recorded, and timely targeted intervention should be given to effectively improve their quality of life.

## 6 Conclusion

This review delves into the risk factors associated with CVT, recent treatment advancements and their influence on patient outcomes. It's found that risk factors for CVT are diverse and are closely related to its outcomes and prognosis. Early identification of risk factors that can be treated and controlled is essential for prevention and early intervention. With regard to treatment, DOACs are as effective and safe as VKAs, suggesting a viable alternative to CVT patients. EVT serves as a second‐line therapy for CVT, and its effectiveness and safety still need further validation and effective devices and technologies are being explored. Also by analyzing the current issues in CVT management, future directions and perspectives for treatment were proposed, highlighting the need for further research to optimize patient care and outcomes.

## Author Contributions

**Wenbo Zuo**: conceptualization, writing – original draft, writing – review and editing. **Shengcai Chen**: methodology, writing – review and editing, writing – original draft. **Jiashuo Lin**: writing – original draft. **Yan Wan**: methodology, supervision, writing – review and editing. **Fei Cao**: supervision. **Bo Hu**: methodology, supervision, funding acquisition, project administration.

## Conflicts of Interest

The authors declare no conflicts of interest.

## Peer Review

The peer review history for this article is available at https://publons.com/publon/10.1002/brb3.70844

## Supporting information




**Supplementary Table**: brb370844‐sup‐0001‐TableS1.docx

## Data Availability

Data sharing not applicable to this article as no datasets were generated or analyzed during the current study.
