# Supplementary material for: Cerebral Venous Thrombosis:Current Status and Challenges
Source: Brain Behav. 2025 Sep 9;15(9):e70844. doi: 10.1002/brb3.70844 (PMC12417975; doi:10.1002/brb3.70844)
Supplement: Supplementary file 1 — Supplementary Table: brb370844‐sup‐0001‐TableS1.docx [file BRB3-15-e70844-s001.docx]

| **Classification** | **Risk factors** | **Subgroup of risk factors** | **Managemant** | **References** |
| --- | --- | --- | --- | --- |
| Short-term factors | Woman-specific | Perinatal period | Standard anticoagulation therapy | (Silvis et al., 2019) |
|  |  | Obesity | Anti-inflammatory treatment for improving coagulation factors + anticoagulation therapy | (Zuurbier et al., 2016) |
|  |  | Oral contraceptives | Standard anticoagulation therapy + regular coagulation monitoring (coagulation FVIII, vWF) | (Abou-Ismail et al., 2020)  (Cosman et al., 2005) |
|  |  | Adenomyosis | Iron supplementation + standard anticoagulation therapy or hysterectomy | (B. Li et al., 2023) |
|  | Infectious | Head and neck infection | Active symptomatic anti-infection treatment + standard anticoagulation therapy | (Karakas et al., 2024) |
|  |  | COVID-19 | Standard anticoagulation therapy + antiepileptic therapy | (Ghosh et al., 2021) |
|  |  | Actinomycetes | Penicillin/amoxicillin +6 months of standard anticoagulation therapy | (Martins Sousa et al., 2022) |
|  |  | Tuberculosis bacteria | Standardized anti-tuberculosis treatment +3 to 6 months of standard anticoagulation therapy | (Q. Li et al., 2023) |
|  |  | Klebsiella pneumoniae | Staged antibacterial regimen + rivaroxaban anticoagulant therapy | (Zhou et al., 2022) |
|  |  | Burkholderia pseudoflora | Surgical resection + ceftazidime/meropenem for 2-6 weeks + cotrimoxazole for 6 months + standard anticoagulation therapy | (Bahuleyan et al., 2022) |
|  |  | Scrub typhus | Doxycycline for 10 days + standard anticoagulation for 6 months | (Das et al., 2021) |
|  |  | Leptospira | 7 days of penicillin + 6 months of standard anticoagulation | (Turhan et al., 2006) |
|  |  | Varicella-zoster virus | Acute intervention with acyclovir + phenytoin + short-term heparin followed by warfarin anticoagulation for 6 months | (Archana et al., 2022) |
|  |  | Echinococcosis | Surgery + antiparasitic drugs (albendazole) + anticoagulant therapy | (Namvar et al., 2023) |
|  | Mechanical | Neurosurgical procedures | Routine anticoagulation therapy is not recommended | (Sturiale et al., 2023)  (Wu & Wang, 2024) |
|  |  | Skull fractures | Routine anticoagulation therapy is not recommended | (Ma et al., 2024) |
|  |  | Indwelling catheter related | Routine anticoagulation therapy is not recommended | (Ren et al., 2020) |
|  |  | Lumbar puncture and spinal anesthesia were performed | Routine anticoagulation therapy is not recommended | (Risi et al., 2024) |
| Long-term factors | Autoimmune | Behçet's syndrome | Active anti-inflammatory therapy + standard anticoagulation therapy | (Bettiol et al., 2023) |
|  |  | APS | Lifelong anticoagulation with warfarin | (Aguiar de Sousa et al., 2011) |
|  |  | Immune thrombotic thrombocytopenia is caused by COVID-19 vaccines | Standard first-line therapy + IVIG + oral anticoagulants to neutralize PF4 antibodies | (Ropper & Klein, 2021)  (Saposnik et al., 2024) |
|  | Metabolic | Elevated FVIII | Standard anticoagulation therapy | (Ken-Dror et al., 2024) |
|  |  | Elevated vWF | Standard anticoagulation therapy | (Rietveld et al., 2019) |
|  |  | Anemia | Hemoglobin supplementation + standard anticoagulation therapy | (Silvis et al., 2020) |
|  |  | Hypereosinophilia | Corticosteroids + anticoagulant therapy | (Song et al., 2021) |
|  | Tumor | Active malignant tumor | Apixaban anticoagulant therapy | (Dinç et al., 2023) |
|  |  | Acute lymphoblastic leukemia | L-asparaginase + dexamethasone + standard anticoagulation therapy | (Alet et al., 2020)  (Ranta et al., 2015) |
|  |  | Myeloproliferative neoplasm | Symptomatic treatment + anticoagulant therapy | (Gangat et al., 2021) |
| Permanent factors | Hereditary | Deficiency of protein S or protein C, resistance to activated protein C and deficiency of antithrombin | Warfarin treatment is not recommended  Initial anticoagulation therapy and thrombosis detection should be carried out for 3 to 6 months | (Middeldorp et al., 2023)  (Saposnik et al., 2024) |
| Other factors |  | Smoking, excessive drinking, dehydration, abuse of androgens, nitrous oxide, sildenafil, Fallot's syndrome, dendrofistulas, venous abnormalities, wasp stings, snake bites, HIV,etc | Actively treat the primary disease + conventional anticoagulant therapy | (Banjongjit et al., 2023; Chang et al., 2022; Cohen et al., 2024; Equiza et al., 2020; Geva et al., 1990; Ghosh et al., 2022; Karti et al., 2017; Lippi & Banfi, 2011; Patil et al., 2024; Wan et al., 2024; Zhang et al., 2024) |

Abbreviations: **APS:**Antiphospholipid syndrome; **FVIII:**Factor VIII; **vWF:** von Willebrand factor; **COVID-19:** Corona Virus Disease 2019 ; **APS:**Antiphospholipid syndrome; **IVIG:** Intravenous immunoglobulin; **PF4:**Platelet factor 4; HIV：Human Immunodeficiency Virus.
